# Kinetics of capillary refill time after fluid challenge

**DOI:** 10.1186/s13613-022-01049-x

**Published:** 2022-08-13

**Authors:** Lisa Raia, Paul Gabarre, Vincent Bonny, Tomas Urbina, Louai Missri, Pierre-Yves Boelle, Jean-Luc Baudel, Bertrand Guidet, Eric Maury, Jeremie Joffre, Hafid Ait-Oufella

**Affiliations:** 1 Service de Médecine Intensive-Réanimation, Assistance Publique-Hôpitaux de Paris, Hôpital Saint-Antoine, 184 rue du Faubourg Saint-Antoine, 75571 Paris cedex 12, France; 2 Sorbonne Université, Paris, France; 3 Service de Santé Publique, Assistance Publique-Hôpitaux de Paris, Hôpital Saint-Antoine, Paris, France; 4 Inserm U970, Paris Research Cardiovascular Center, Paris, France

**Keywords:** Sepsis, Kinetics, Fluid challenge, Capillary time refill, Intensive care medicine

## Abstract

**Background:**

Capillary refill time (CRT) is a valuable tool for triage and to guide resuscitation. However, little is known about CRT kinetics after fluid infusion.

**Methods:**

We conducted a prospective observational study in a tertiary teaching hospital. First, we analyzed the intra-observer variability of CRT. Next, we monitored fingertip CRT in sepsis patients during volume expansion within the first 24 h of ICU admission. Fingertip CRT was measured every 2 min during 30 min following crystalloid infusion (500 mL over 15 min).

**Results:**

First, the accuracy of repetitive fingertip CRT measurements was evaluated on 40 critically ill patients. Reproducibility was excellent, with an intra-class correlation coefficient of 99.5% (CI 95% [99.3, 99.8]). A CRT variation larger than 0.2 s was considered as significant. Next, variations of CRT during volume expansion were evaluated on 29 septic patients; median SOFA score was 7 [5–9], median SAPS II was 57 [45–72], and ICU mortality rate was 24%. Twenty-three patients were responders as defined by a CRT decrease  > 0.2 s at 30 min after volume expansion, and 6 were non-responders. Among responders, we observed that fingertip CRT quickly improved with a significant decrease at 6–8 min after start of crystalloid infusion, the maximal improvement being observed after 10–12 min (−0.7 [−0.3;−0.9] s) and maintained at 30 min. CRT variations significantly correlated with baseline CRT measurements (*R* = 0.39, *P* = 0.05).

**Conclusions:**

CRT quickly improved during volume expansion with a significant decrease 6–8 min after start of fluid infusion and a maximal drop at 10–12 min.

**Supplementary Information:**

The online version contains supplementary material available at 10.1186/s13613-022-01049-x.

## Introduction

Peripheral tissue hypoperfusion has been identified as a powerful predictor of poor outcomes in patients suffering from severe conditions, including sepsis [1], cardiac arrest [2] or cardiogenic shock [3]. International guidelines highlighted that the evaluation of peripheral tissue perfusion is of paramount importance to identify shock patients [4]. Peripheral tissue perfusion could be evaluated at the bedside with easy-to-use and easy-to-learn tools, either semi-quantitative such as the mottling score, or quantitative, including skin temperature and the capillary refill time (CRT) [5].

The capillary refill time (CRT) measures the time necessary for the skin to return to baseline color after applying pressure on soft tissue (generally the fingertip). Interrater variability of CRT was weak in non-trained physicians [6] but is very good after standardization in centers expert in peripheral tissue perfusion evaluation [1, 7]. CRT is a valuable tool to assess the severity of an acute illness at both early and late stages. In the emergency ward, persistent prolonged fingertip CRT (> 3 s) is associated with more severe organ failure, more frequent use of organ support therapy and ultimately, a higher in-ICU mortality [8]. In the intensive care unit, in a mixed critically ill population, Lima et al. have reported that a prolonged CRT (> 4.5 s on the index finger) was associated with hyperlactatemia and high SOFA score [9]. Finally, in septic shock patients, persistent prolonged finger CRT after resuscitation is predictive of 14-day mortality, with an Area Under Curve of 84%. A 2.4 s threshold value predicted mortality with good sensitivity (82%, 95% CI [60–95]) and specificity (73%, 95% CI [56–86]) [1]. More recently, the ANDROMEDA-SHOCK trial provided convincing evidence that CRT can be used to guide treatment and resuscitation [10]. In septic shock patients, a strategy based on CRT monitoring led to more important organ failure recovery than an approach based on lactate clearance associated with improved survival. In this trial, the CRT was measured every 30 min but the precise kinetics of CRT after therapeutic intervention is not known. This study aimed to analyze the kinetics of CRT variations after a fluid challenge in sepsis patients.

## Methods

### Study design and measurements

We conducted a prospective observational study in an 18-bed ICU in a tertiary teaching hospital. During 3 months, all consecutive patients, older than 18 years, to whom the attending intensivist decided to administer a volume expansion were screened. Patients with a prolonged CRT (> 2.5 s) within the first 24 h of ICU admission were included. In addition, to limit confounders, we focused on patients with sepsis with or without shock according to the Third International Consensus Definitions [11]. Patients with dark skin for whom accurate clinical evaluation of CRT was not possible were excluded. During the kinetic study, fingertip CRT was measured twice and the mean value was recorded. CRT was measured every 2 min for 20 min and finally at 25 and 30 min by one single physician. CRT measurements started at the same time that crystalloid infusion was initiated. As previously reported and standardized by our group, CRT was measured by applying firm pressure to the distal phalanx of the index finger for 15 s. The pressure applied was just enough to remove the blood at the tip of the physician’s nail, which was illustrated by the appearance of a thin white distal crescent (blanching) under the nail. A chronometer recorded the time for the return to the baseline color [1]. Volume expansion was standardized with the infusion of 500 mL of crystalloids (saline or ringer lactate) over 15 min.

### Patient management and data collection

Patients were admitted directly from the emergency department or medical wards. Circulatory support was guided by our local protocol, adapted from international guidelines [12]. Initial therapeutic management includes antibiotic administration, fluid infusion (30 mL/Kg), norepinephrine infusion to maintain a mean arterial pressure (MAP)  > 65 mmHg and infection source control when available. All patients were investigated with transthoracic echocardiography (Vivid 7 Dimension’06, GE Healthcare^®^) to assess left ventricular function, volemia and cardiac output. Repetitive transthoracic echocardiography was performed routinely during acute circulatory failure management. A fluid infusion was decided by the physician in charge of the patient and was based on several parameters as indicated by international guidelines [13].

General characteristics of the patients were recorded: demographic data, diagnoses, severity of illness evaluated by the Sequential Organ Failure Assessment (SOFA) score [14] and Simplified Acute Physiology Score II (SAPS II) [15]. We collected MAP, heart rate (HR) and cardiac index. Tissue and organ perfusion were assessed through arterial lactate level, urinary output, mottling score, skin temperature, and fingertip CRT.

### Statistical analysis

Patient characteristics were summarized as mean ± standard deviation, median (25th–75th percentiles) for skewed distributions and percentages as appropriate. Differences between groups were compared using the Mann–Whitney test or Wilcoxon’s test. Correlations were computed using Pearson’s formula.

CRT was measured to the nearest tenth of a second. Two measurements were obtained for each patient. Reproducibility (variation due to measurement method) was assessed by the intra-class correlation coefficient (ICC), defined as the ratio between-individual variance to the sum of between- and within-individual variance. An ICC close to 1 shows that all variance is explained by between-individual variation and not by within-individual the total variance. ICC was obtained from random-effect ANOVA (R package multilevel). Confidence intervals were obtained by (patient-level) bootstrap.

Statistical significance was set at *P* < 0.05. All analyses were made using the R software (v 2.12.0; http://cran.r-project.org).

The ethical committee of the French Intensive Care Society (FICS) approved the protocol (*CE SRLF 22-009).* (*CE SRLF 22-009).* This is an observational study without any specific intervention. Volume expansion was decided by the physician in charge of the patient and repetitive CRT measurements were recorded. Repetitive CRT measurements during fluid infusion are routinely performed in our ICU.

## Results

### Intra-reader reproducibility of the Capillary Refill Time

**Fig. 1:**
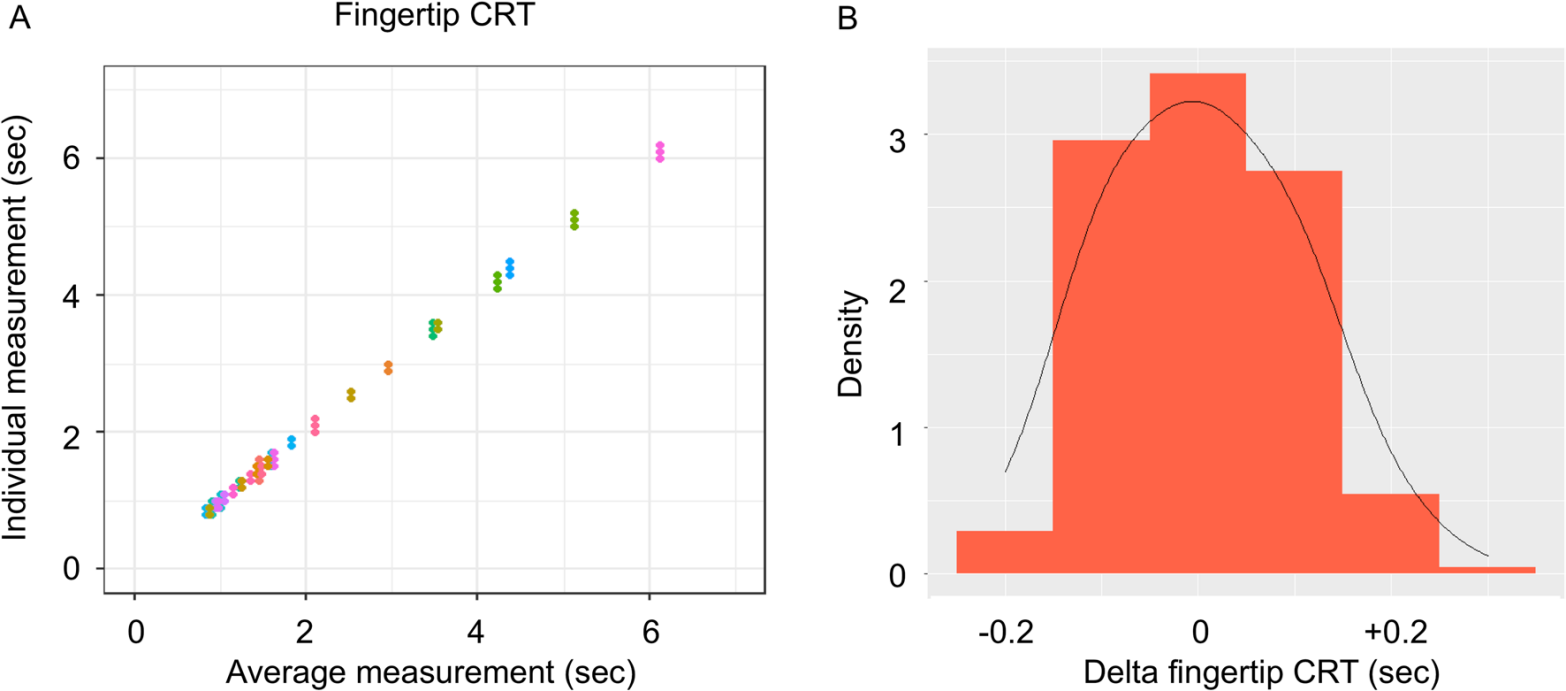
**A** Individual and patient-averaged repeated fingertip CRT measurements at a single timepoint (seconds). Data are color-coded by patient. **B** Distribution of variations of fingertip CRT measurements (seconds)

We have previously reported an excellent inter-reader reproducibility of CRT measurements [1]. Given that one physician monitored CRT kinetics for each patient, we first analyzed the intra-reader reproducibility of CRT with two physicians who participated in this study, both having experience in CRT measurements and peripheral tissue evaluation. Forty critically ill patients were prospectively included in this first study and 3 fingertip CRT measurements were done by each physician. The reproducibility of fingertip CRT was high (Fig. 1A). The ICC was 99.5% (CI 95% [99.3, 99.8]), suggesting that 0.5% of the total variance was due to measurement and the rest to patient peripheral perfusion change. Intra-reader standard deviation of the fingertip CRT was 0.07 s. Therefore, we assumed that a CRT variation larger than 0.2 s was significant [16] (Fig. 1B).

### Studied population

Next, we investigated prospectively kinetics of fingertip CRT variations induced by a fluid challenge. Over 3 months, 68 patients received fluid expansion during the first 24 h of ICU admission. 37 patients were excluded, 18 patients with no sepsis, 8 patients, because accurate CRT measurements were not possible (dark skin), 11 patients with CRT  < 2.5 s, leaving 31 patients for the study. Among them, 25 patients were responders as defined by a CRT decrease  > 0.2 s at 30 min after fluid challenge, and 6 were non-responders. Finally, 2 responders were excluded, because vasopressor dose was increased during the fluid challenge leaving 23 responders for analysis (Additional file 1). Baseline characteristics are summarized in Table 1. All patients had sepsis without (*N* = 15/29) or with shock (*N* = 14/29). The main causes were pneumonia (41%) and abdominal infection (27%). Disease severity was more pronounced in responders with higher SOFA score at admission (5 [4–6] versus 7 [5–10], *P* = 0.03) and more frequent use of invasive mechanical ventilation (17% versus 61%, *P* = 0.05). Baseline fingertip CRT was longer in responders (2.8 [2.5–3.4] versus 3.8 [2.6–4.1] sec, *P* = 0.02). In ICU mortality rate was 24%.

**Table 1:** Baseline characteristics of studied population. Parameters and SOFA (Sequential Organ Failure Assessment) were reported at inclusion

	Total *N* = 29	Non-responders *N* = 6	Responders *N* = 23	*P* value
Age, years	67 [57–71]	67 [57–68]	67 [57–71]	0.85
Male gender	21 (72.4)	3 (50)	18 (78)	0.30
Body mass index, kg/m^2^	25 [22–28]	25 [23–28]	25 [19–29]	0.92
SAPS II	57 [45–72]	46 [44–47]	61 [50–78]	0.10
SOFA	7 [5–9]	5 [4–6]	7 [5–10]	**0.03**
Comordibities				
Cardiovascular disease	5 (17)	1 (17)	4 (17)	> 0.99
Hypertension	18 (62)	2 (33)	16 (69)	0.24
Cirrhosis	0	0	0	–
Diabetes mellitus	12 (41)	1 (17)	11 (48)	0.36
Chronic kidney disease	5 (17)	0 (0)	4 (17)	0.27
Malignancy	12 (41)	3 (67)	9 (39)	0.63
Sepsis				
Proven	21 (72)	4 (67)	17 (74)	
Suspected	8 (35)	2 (33)	6 (26)	0.72
Heart rate, bpm	109 [95–127]	124 [89–152]	106 [95–125]	0.34
MAP, mmHg	71 [67–82]	74 [70–79]	71 [68–83]	0.94
Cardiac index, mL/min/m^2^	3.4 [2.7–4.5]	3.4 [2.6–4.4]	3.4 [2.6–4.6]	0.84
CRT fingertip, sec	3.4 [2.5–4.0]	2.8 [2.5–3.4]	3.8 [2.6–4.1]	**0.02**
Mottling score	2 [1–3]	1.5 [0.75–3.25]	2 [1–3]	0.51
Skin temperature, °C	29.8 [29.2–31.6]	29.8 [28.8–31]	29.9 [29.2–31.9]	0.89
Mechanical ventilation, *n* (%)	15 (52)	1 (17)	14 (61)	**0.05**
Crystalloid infused before inclusion (mL)	1500 [1000–2500]	1500 [1000–1800]	1500 [1000–2200]	0.67
Norepinephrine				
Yes, *n* (%)	14 (48)	2 (33)	12 (52)	0.41
Dose, μg/kg/min	0 [0–0.40]	0 [0–0.38]	0.10 [0–0.40]	0.53
Biological parameters				
Arterial pH	7.31 [7.26–7.44]	7.29 [7.10–7.40]	7.32 [7.27–7.44]	0.36
Arterial lactate, mmol/L	2.4 [1.4–3.3]	2.3 [1.3–3.4]	2.4 [1.3–3.8]	0.80
Leukocyte count, G/L	13.4 [6.6–18.8]	10.6 [5.5–18.6]	13.4 [7.0–19.3]	0.72
Hemoglobin, g/dL	13.1 [10.3–13.8]	11.7 [9.2–13.7]	13.3 [11–13.9]	0.29
Platelet count, G/L	172 [105–233]	161 [107–289]	172 [105–233]	0.84
Serum creatinin, μmol/L	127 [96–189]	104 [99–117]	131 [84–241]	0.18
Sodium, mmol/L	136 [130–139]	137 [130–140]	136 [129–139]	0.94
Protidemia, g/L	58 [49–62]	59 [49–62]	55 [49–63]	0.98
Bilirubin, μmol/L	14 [10–18]	15 [9–27]	14 [10–19]	0.85
C reactive protein, mg/L	137 [30–390]	161 [75–282]	100 [4–398]	0.76
Procalcitonin, ng/L	10.3 [2.9–65]	13.5 [5.7–87.8]	10.5 [1.2–63.6]	0.48
Death in ICU, *n* (%)	7 (24)	0 (0)	7 (30)	0.12

SAPS II (Simplified Acute Physiology Score) was calculated within 24 h of admission. Values are given as median (25th–75th percentiles) and percentage according to data distribution. Comparisons were done using non-parametric Mann Whitney test. Patients’ characteristics

*ICU* intensive care unit, *MAP* mean arterial pressure, *SAPS* Simplified Acute Physiology Score, *SOFA* Score SOFA (Sequential Organ Failure Assessment), *CRT* capillary refill time

Bold values indicate statistically differences between groups

In responders, 30 min after infusion the heart rate significantly decreased, while systolic blood pressure increased, with a trend to an increase in cardiac index. No difference in mottling score was observed between the two timepoints (Table 2).

**Table 2:** Global hemodynamic and tissue parameters at baseline and 30 min after fluid infusion parameters

Parameters	Baseline	30 min	*P* value
Heart rate, bpm	106 [95–125]	103 [90–124]	**0.01**
Blood pressure			
Systolic, mmHg	100 [94–123]	109 [95–131]	**0.05**
Diastolic, mmHg	59 [55–69]	62 [56–67]	0.51
Mean, mmHg	71 [68–83]	78 [69–86]	0.11
Norepinephrine, μg/kg/min	0.10 [0–0.40]	0.10 [0–0.40]	1
Cardiac index, L/min/m2	2.8 [2.2–3.7]	3.4 [2.3–4.3]	0.12
Capillary refill time	3.8 [2.6–4.1]	2.5 [1.5–3.4]	** < 0.0001**
Mottling score	2 [1–3]	2 [0–3]	0.13

Values are given as median [25th–75th percentiles]. Comparisons were done using paired non-parametric test. Global hemodynamic and tissue perfusion parameters baseline and 30 min after fluid infusion in responders (*N* = 23)

Bold values indicate statistically differences between groups

### Fingertip CRT kinetics

**Fig. 2:**
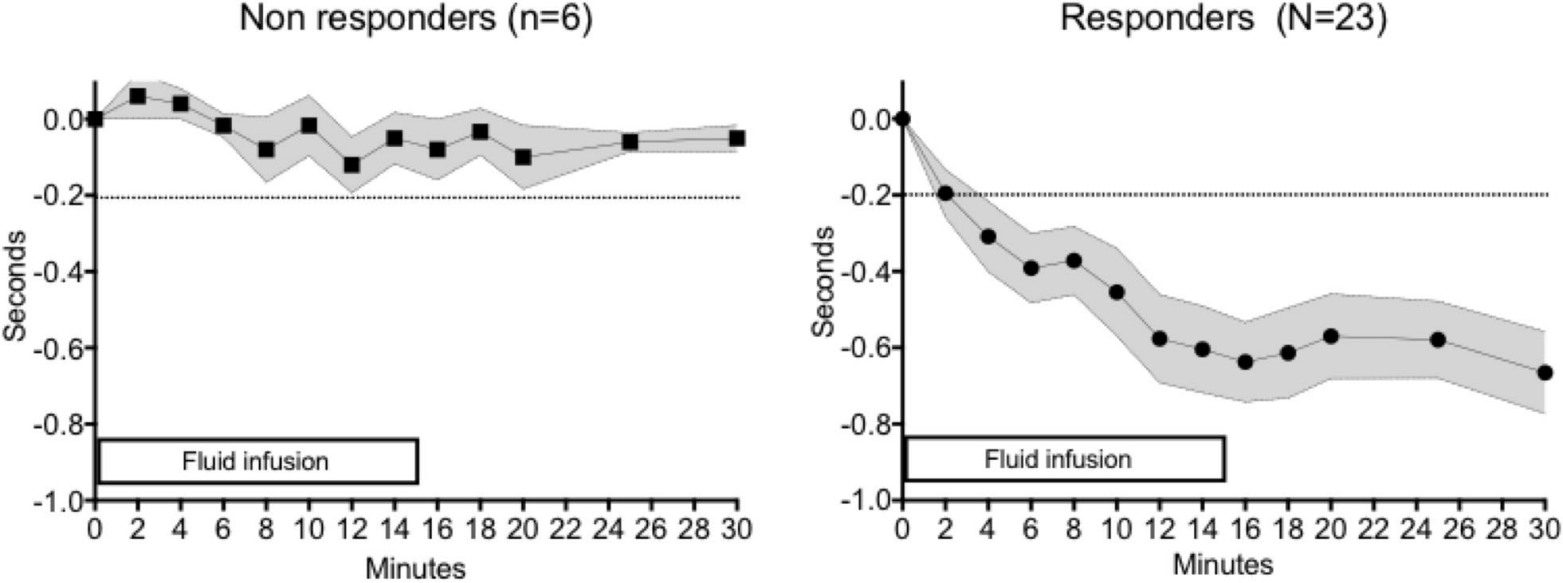
Kinetics of the variations of fingertip CRT overtime in non-responders (left) and in responders (right). Measurements started upon start of crystalloid infusion. Data were expressed as mean ± SD

Fingertip CRT monitoring was started when the crystalloid infusion was initiated during a total 30 min period. We observed that fingertip CRT significantly decreased very quickly. Indeed, variations larger than 0.2 s were observed 6–8 min after the initiation of crystalloid infusion. Maximal fingertip CRT drop was observed 10–12 min after the start of infusion (−0.7 [−0.3;−0.9] sec) and this drop persisted until the end of the 30 min monitoring (Fig. 2A, B). The variations of the fingertip CRT correlated positively with the baseline values (*R* = 0.39, *P* = 0.05).

## Discussion

In sepsis patients we showed that fingertip CRT quickly improved after crystalloid infusion was initiated, with a significant decrease at 6–8 min, the maximal improvement being observed after 10–12 min and persisting at 30 min. CRT variations significantly correlated with baseline CRT measurements.

In pediatric wards, CRT has been mainly used as a triage tool to identify the most severe children suffering from infectious diseases, such as pneumonia, gastroenteritis and malaria [17, 18]. After initial resuscitation, a prolonged CRT identified sepsis patients with poor outcomes in both the emergency ward [8] and ICU [1]. More recently, Hernandez et al. reported that index tip CRT “normalization” at H6 after resuscitation was associated with a good prognosis [19]. Our group also found that improvement in peripheral tissue hypoperfusion is strongly associated with 14-day survival in septic shock patients [20]. Finally, in the ANDROMEDA-SHOCK trial, monitoring CRT every 30 min was used to guide early therapeutic strategy in septic shock patients, and suggested a benefit when compared to a lactate clearance guided approach [10]. Choosing a 30 min timepoint was mainly based on physician clinical experience, but the accurate timing of CRT variations during volume expansion in critically ill patients remained unknown. Despite no detailed timing for measurements, Jacquet-Lagrèze et al. reported a decrease in fingertip CRT after passive leg raising [21], suggesting that variations of CRT during fluid infusion may be rapid. We found that the CRT quickly improved with maximal response observed at 10–12 min after fluid expansion initiation.

Decreased CRT following fluid infusion may reflect improved microvascular perfusion as previously reported by Ospina-Tascon with videomicroscopy in the sublingual area during the early phase of resuscitation [22]. Several mechanisms may be responsible for improved microvascular perfusion, such as an attenuation of sympathetic-induced vasoconstriction or increased cardiac output. Here, we observed lower heart rate after fluid challenge supporting a reduction of sympathetic activation after volume expansion and a trend to increased cardiac index. Variability in cardiac index measurements using echocardiography [23] may explain, at least in part, the absence of significant difference between baseline and 30 min timepoints. Finally, the normalization of peripheral tissue hypoperfusion despite the absence of cardiac index increase following fluid infusion could also be due to blood dilution and improved microvascular rheology [24].

Different thresholds have been previously proposed to define prolonged CRT. Schriger et al. proposed 4.5 s while comparing subjects before and after fingertip exposure to cold water, which is far from the clinical setting [25]. We selected patients with fingertip CRT  > 2.5 s based on our previous study on selected septic shock patients reporting that the index CRT cutoff at 2.4 s was a strong predictor of 14-day mortality[1] [5]. We report in this study a positive correlation between baseline CRT and variations after fluid challenge. In other words, the higher the baseline CRT, the higher the decrease after volume expansion.

In previous works, our group found that a decrease in mottling score 6 h after resuscitation is associated with better 14-day survival [20, 26]. Here, in responders, mottling score did not significantly change 30 min after fluid infusion, whereas CRT dropped. Such observation suggests that the mottling score is an appropriate triage tool but could not be used as a monitoring tool to guide rapid therapeutic intervention.

Our study has several limitations. It is a monocentric study, and results need to be confirmed in a larger population. CRT was monitored during a total of 30 min but only 15 min after the end of volume expansion and we cannot excluded delayed CRT improvement in the non-responders. In our study, criteria for volume expansion were not standardized and the intervention was decided by the physician in charge of the patient. Therefore, the evaluation of fluid responsiveness before the challenge was not recorded. In addition, CRT kinetics were measured by physicians with expertise in the evaluation of peripheral tissue perfusion and training is of paramount importance to perform accurate monitoring of CRT.

## Conclusions

Fingertip CRT quickly improved during volume expansion in over 80% of septic patients, with a significant decrease as soon as 6–8 min after start of fluid expansion, and a maximal improvement at 10–12 min.

## Supplementary Information

**Additional file 1. **Flow chart of studied population.

## Data Availability

The data sets used and/or analyzed during the current study are available from the corresponding author on reasonable request.

## Abbreviations

CRT: Capillary refill time
ICC: Intra-class correlation coefficient
ICU: Intensive care unit
MAP: Mean arterial pressure
SOFA: Sequential organ failure assessment
SAPS II: Simplified acute physiologic score II
