# Supplementary material for: Kinetics of capillary refill time after fluid challenge
Source: Ann Intensive Care. 2022 Aug 13;12:74. doi: 10.1186/s13613-022-01049-x (PMC9375797; doi:10.1186/s13613-022-01049-x)
Supplement: Supplementary file 1 — Additional file 1. Flow chart of studied population. [file 13613_2022_1049_MOESM1_ESM.pptx]

## Slide 1
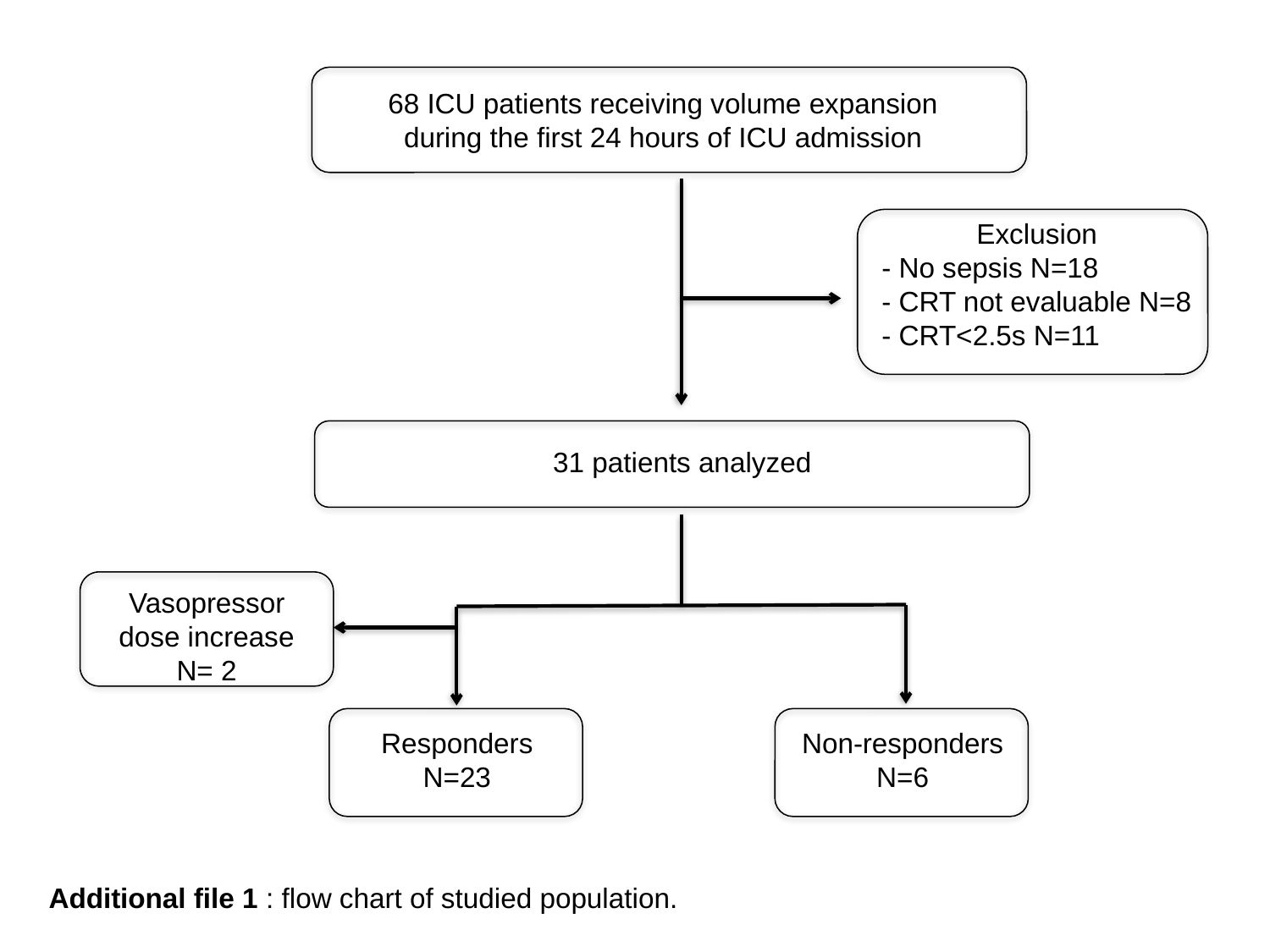

68 ICU patients receiving volume expansion during the first 24 hours of ICU admission
Exclusion
- No sepsis N=18
- CRT not evaluable N=8
- CRT<2.5s N=11
31 patients analyzed
Vasopressor dose increase
N= 2
Responders
N=23
Non-responders
N=6
Additional file 1 : flow chart of studied population.
